# Hyaluronic Acid: Molecular Mechanisms and Therapeutic Trajectory

**DOI:** 10.3389/fvets.2019.00192

**Published:** 2019-06-25

**Authors:** Ramesh C. Gupta, Rajiv Lall, Ajay Srivastava, Anita Sinha

**Affiliations:** ^1^Toxicology Department, Breathitt Veterinary Center, Murray State University, Hopkinsville, KY, United States; ^2^Vets Plus, Inc., Menomonie, WI, United States

**Keywords:** hyaluronic acid, hyaluronan, osteoarthritis, viscosupplementation, wound healing, ophthalmic diseases, adjuvant therapy, cancer therapy

## Abstract

Hyaluronic acid (also known as hyaluronan or hyaluronate) is naturally found in many tissues and fluids, but more abundantly in articular cartilage and synovial fluid (SF). Hyaluronic acid (HA) content varies widely in different joints and species. HA is a non-sulfated, naturally occurring non-protein glycosaminoglycan (GAG), with distinct physico-chemical properties, produced by synoviocytes, fibroblasts, and chondrocytes. HA has an important role in the biomechanics of normal SF, where it is partially responsible for lubrication and viscoelasticity of the SF. The concentration of HA and its molecular weight (MW) decline as osteoarthritis (OA) progresses with aging. For that reason, HA has been used for more than four decades in the treatment of OA in dogs, horses and humans. HA produces anti-arthritic effects via multiple mechanisms involving receptors, enzymes and other metabolic pathways. HA is also used in the treatment of ophthalmic, dermal, burns, wound repair, and other health conditions. The MW of HA appears to play a critical role in the formulation of the products used in the treatment of diseases. This review provides a mechanism-based rationale for the use of HA in some disease conditions with special reference to OA.

## Introduction

In 1934, Karl Meyer and John Palmer isolated for the first time a glycosaminoglycan (GAG) from the vitreous humor of the bovine eye and named it “hyaluronic acid” (derived from hyaloid [vitreous] and uronic acid). The term “hyaluronan” was introduced in 1986 to conform to polysaccharide nomenclature. Subsequently, it was found in other organs (joints, skin, rooster comb, human umbilical cord, etc.) and tissues (connective, epithelial, and nervous). Hyaluronic acid (HA) is also produced via microbial (*Streptococcus zooepidemicus, Escherichia coli, Bacillus subtilis*, and others) fermentation (1–3), and its molecular weight (MW) is reported to be controlled by UDP-N-acetylglucosamine concentration (4). In both vertebrates and bacteria, its chemical structure is identical (5, 6). Most cells in the body have the capability to synthesize HA during some point of their cell cycles, implicating its function in several fundamental biological processes (7–10). HA is a major component of the extracellular matrix (ECM) and is normally present in mammalian bone marrow, articular cartilage, and synovial fluid.

The first therapeutic injections of HA in animal joints were performed on track horses for traumatic arthritis. This treatment proved effective and since then it has been widely used in veterinary medicine (11, 12). Currently, elastoviscous HA solutions and its derivatives (such as Hylans) are commonly used in animals for treatment of arthritic pain. HA is reported to be a unique biomolecule because its biological functions can be attributed to its physico-chemical properties and to its specific interactions with cells and ECM (7, 10, 13). HA has recently become more widely accepted in the armamentarium of therapies for OA pain (14, 15). In humans, HA has been used since the 1970s for treating joint pain and other health conditions (8, 10, 16–22).

This review describes physico-chemical and rheological properties, cellular and molecular mechanisms in pharmacological and therapeutic effects in health and disease conditions, and toxicity and safety considerations of HA.

## Physico-Chemical Properties and Physiological Functions

**Figure 1:**
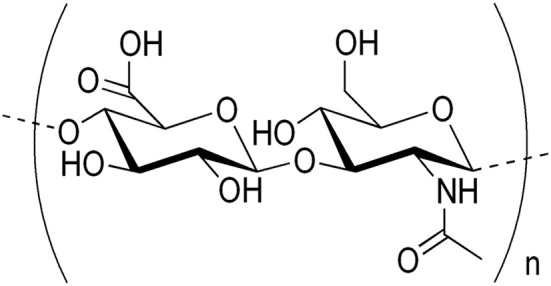
Structural formula of hyaluronic acid (HA).

Hyaluronic acid (HA) is a naturally occurring non-sulfated glycosaminoglycan (GAG) non-protein compound with distinct physico-chemical properties of repeating β-1,4-D-glucuronic acid and β-1,3-*N*-acetylglucosamine units (10, 16, 23–25). The structural formula of HA is shown in Figure 1. HA has excellent viscoelasticity, high moisture retention capacity, high biocompatibility, and hygroscopic properties (16, 26). At a concentration as low as 0.1%, HA chains can provide high viscosity (23). By having these properties, HA acts as a lubricant, shock absorber, joint structure stabilizer, and water balance- and flow resistance-regulator (3, 27, 28).

A person with an average weight of 70 kg has about 15 g of HA, which is present in joints, skin, eyes and other organs and tissues (connective, epithelial, and neural) of the body (7, 29, 30). Out of 15 g total HA, 5 g turns over daily (31). The greatest amount of HA is present in the skin (about half of the total HA (32), synovial fluid (33), the vitreous body (34), and the umbilical cord (35). HA is an important constituent of ECM and contributes to cell proliferation, migration, and morphogenesis (10, 36–38). HA also occurs within cells and it has been reported to have roles inside the cell (37, 39). Within the joint cavity, HA molecules are predominately synthesized by type B synoviocytes. HA (a polymer of disaccharides) can be 25,000 disaccharide repeats in length with a MW of 5,000–20,000,000 Da.

HA is synthesized by hyaluronan synthase (HAS), of which vertebrates have three isozymes (HAS-1, HAS-2, and HAS-3). These three HAS isozymes produce different size HA polymers and are differentially regulated by transcriptional, translational and post-translational levels, including alternative splicing, sub-cellular localization and epigenetic processes. These isoenzymes lengthen HA by repeatedly adding glucuronic acid and *N*-acetylglucosamine to the nascent polysaccharide. The three genes are located on three different chromosomes, even though they have 50–71% identity. They occur at 19q13.4, 8q24.12, and 16q22.1, respectively (40). HA is catabolized by hyaluronidases, and the MW of HA in cartilage is reported to decrease with age (8, 41–44).

HA binds to ECM molecules and cell surface receptors, thereby regulating cellular behavior via control of the tissue's macro- and micro-environments (7). In an *in vitro* study, Sommarin and Heinegård (45) investigated the interaction between HA and exogenous [^35^S]sulfate-labeled cartilage proteoglycans (PGs) at the calf articular-cartilage chondrocyte cell surface. Findings revealed that PGs interact with HA receptors at the cell surface in the HA-binding region. The bound ^35^S-labeled PGs are located at the cell surface, and only small proportions of the PGs are internalized. HA can bind to three main classes of cell surface receptors: (1) CD44 (a membrane glycoprotein), (2) receptor for hyaluronate-mediated motility (RHAMM), and (3) Intercellular Adhesion Molecule 1 (ICAM-1), which perform different functions (7, 46). CD44 is the most widely distributed cell surface receptor recognized for HA binding (8, 10, 47–49). CD44 interacts with a number of other ligands including osteopontin, collagens and matrix metalloproteinases (MMPs). HA may inhibit signal transduction through CD44 (50, 51) and RHAMM (52, 53) HA receptors. It is reported that higher- and lower- MW HA have distinct molecular and cellular mechanisms and diverse biological effects through interaction with CD44 receptors (54–56). CD44-mediated signaling affects both chondrocyte survival pathways as well as apoptotic (chondroptotic) pathways. Fragments of HA produced in free radical processes have the potential to augment the production of nitric oxide (NO) in a CD44-dependent mechanism. In regard to defining functional chondrocyte CD44, future studies need to include analysis of the variant CD44 isoforms expression, phosphorylation, cytoskeletal interactions, occupancy, and turnover. In addition to these receptors, two other receptors have been identified for HA binding: (1) lymphatic vessel endothelial hyaluronan receptor (LYVE-1), and (2) hyaluronic acid receptor for endocytosis (HARE), also known as Stabilin-2 (40).

Physiological roles of HA are well-characterized in body tissues and fluids (7, 27, 57). In general, HA may be involved in various cellular interactions (cell differentiation, proliferation, development, and recognition) and physiological functions (lubrication, hydration balance, matrix structure, and steric interactions) (58). By having unique rheological properties and being a constituent of GAG and articular cartilage, the physiological roles of HA are well-explained in normal structure and function of joints. The physiological relevance of HA is not only recognized in healthy and OA joints (14, 57–60), but also in other tissues and health conditions (7, 8, 10, 36, 61, 62).

Physiological and pharmacological mechanisms involved in effects of HA in SF are summarized in Table 1.

**Table 1:** Physiological and pharmacological mechanisms and effects of HA in synovial fluid.

**Mechanism and effects**	**References**
**PHYSIOLOGICAL**	
1.Maintenance of viscoelasticity	(23, 63)
2.Restores rheological properties and metabolism of fibroblasts	(25)
3.Maintenance of lubrication	(63, 64)
**PHARMACOLOGICAL**	
1.Scavenges ROS/RNS and exerts antioxidative effect	(65)
2.Exerts anti-inflammatory effect	(57, 66)
3.Reduces production of MMPs (MMP-1, MMP-3, and MMP-13)	(67, 68)
4.Reduces production and activity of IL-1β, and other pro-inflammatory mediators	(67)
5.Inhibits synthesis of PGE_2_ and bradikinin	(69)
6.HA mitigates synovial hypertrophy and increases the number of synovial fibroblast-like cells, while decreasing macrophages, lymphocytes, mast cells and adipocytes	(25)
7.Regulates fibroblast proliferation	(16)
8.Inhibits migration and aggregation of leukocyte and macrophages	(11)
9.Alters behavior of immune cells	(17, 70)
10.Enhances synthesis of chondrocytes, HA, and proteoglycan	(16, 71–74)
11.Improves viscoelasticity and enhances lubricating potential	(27, 28, 59, 64, 75, 76)
12.Improves joint function, mobility, and reduces stiffness	(64, 77)
13.HA interacts with HA receptors on or around the free nerve endings, thereby producing analgesia	(28, 78)

All these physico-chemical properties of HA have been shown to be MW-dependent (10, 25, 79, 80). Aviad and Houpt (81) suggested that the beneficial effect of injected HA in joint disease, such as OA, may be due to pharmacological effects rather than to physico-chemical properties (such as SF viscosity, HA concentration, HA MW, and the rate of synthesis). Taken together, findings suggest that the beneficial effects of HA may be due to both rheological properties and pharmacological mechanisms.

## Pharmacokinetics of HA

Pharmacokinetic data of HA in animals and humans are sparse. Balogh et al. (82) reported for the first time pharmacokinetics (absorption, distribution and excretion) of higher-MW ^99m^technetium HA (^99m^Tc-HA) in Wistar rats (150–200 g each) and Beagle dogs (10–15 kg each) after oral administration. The MW of this preparation was in the range of HA similar to that used in some dietary supplements. All tissues examined showed incorporation of radioactivity from ^99m^Tc-HA starting at 15 min and persisting for 48 h. The whole body scintigraphs and close-ups of the ventral chest region showed non-alimentary radioactivity from ^99m^Tc-HA concentrated in joints, vertebrae and salivary glands 4 h after administration. HA, which is known to have an affinity for connective tissues, exhibited accumulation of radioactivity after oral administration. Balogh et al. (82) also noted that the appearance of radioactivity in tissues before blood suggested that HA was delivered to tissues via a non-blood transport system. Lymphatic uptake and transport could explain these findings. It is also known that transport of higher-MW HA into and out of synovial spaces occurs via lymphatics (83–85) and its normal presence in blood and other fluids (83) provides mechanistic support for the appearance of ingested HA in connective tissues (82). In a number of studies, the distribution of intravenously (IV) administered HA has been studied using ^14^C, ^3^H, ^125^I, and other isotopes as tracers (86–88). In a rat model (adult male Wistar rats weighing 280–350 g each), Svanovsky et al. (89) determined the difference in the biodistributional pathways of ^111^In-labeled diethylenetriamine pentaacetic acid-HA (^111^In-DTPA-HA) molecule of three different MWs (10, 100, and 450 kDa). The study revealed that 50–54% for 10 and 100 kDa, and 80% for 450 kDa of the administered dose of radiolabel HA was present in the liver after 5 min. The elimination of radiolabel was mostly renal and in lower-MW form. Radioactivity remained in liver throughout the 72 h experimental period. Authors concluded that a difference in the biodistribution of 450 kDa and lower-MW radiolabelled molecules was found. Higher amounts of radiolabel were taken up by the liver when the 450 kDa molecule was used, and lower-MW fractions were found in the urine, which could have been products of non-enzymatic cleavage. In an *in vitro* study, Eriksson et al. (90) confirmed that uptake of radiolabelled HA occurred in the liver endothelial cells, and the same cells degraded the HA into lower-MW HA products.

In an experimental study, Engström-Laurent and Hellström (91) determined the concentration of circulating HA in male Sprague-Dawley rats (weighing 300–350 g each) after either the liver or the kidneys had been excluded from the systemic circulation. The rate of increase was more rapid in the animals with ligated hepatic vessels compared to those with ligated renal vessels. This and other studies suggested that both renal and hepatic systems are important for the removal of HA from the blood (83).

In a dialysis study in rats, Breborowicz et al. (92) found that 25% of the administered HA (10 mg/dL; MW 1,800,000–2,400,000) was absorbed over a period of 8 h, suggesting significant absorption of HA from peritoneal interstitium to bloodstream.

Pierce (93) revealed elevated levels of HA in serum after its oral administration in horses. Therapeutic efficacy of HA against lameness was found to be greater with oral than IV administration. Furthermore, HA administered intra-articularly (IA) dissipates out of the joint within 14–18 h. HA diffuses out into surrounding tissues via the bloodstream, circulating throughout the body, and is rapidly eliminated.

In normal adult humans and rabbits, Fraser et al. (94) determined the plasma elimination half-life of HA in between 2.5 and 5.5 min after injection of [^3^H]HA with higher-MW. Reed et al. (95) also reported the half-life of circulating HA in between 3 and 5 min in rabbits injected with [^3^H]HA subcutaneously. The daily turnover of HA in the circulation was estimated to be at least 150 mg. Elimination of HA was predominantly extra-renal, with the upper MW limit for renal excretion being 25,000 Da. Findings revealed that the liver removes ~90% of the circulating HA and the remainder is removed by the spleen within 24 h. HA can be catabolized by three hyaluronidases (HYAL) (96): (1) HYAL1 is associated with lysosomes, which degrades the HA into tetrasaccharides, (2) HYAL2 degrades HA of higher-MW into products of 20 kDa (97), and (3) details of HAYL3 have yet to be elucidated (98). HA in the amounts currently used for therapeutic purposes does not appear to accumulate significantly in the circulation.

## Pharmacotherapeutics of HA

### Hyaluronic Acid (HA) in Osteoarthritis (OA)

#### OA Background

OA is an inflammatory disease of diarthrodial joints characterized by chronic and progressive cartilage degeneration, osteophyte formation, subchondral sclerosis, hypertrophy of bone at the margin, and changes in the synovial membrane (42, 99). With age-related OA, the articular cartilage is subject to significant structural, mechanical, and matrix changes consisting of mild fibrillation of the articular surface and a decrease in PG monomer size and aggregation (100). OA is a disease of the entire joint affecting the articular cartilage, subchondral bone, synovial capsule and membrane, and the periarticular tissues (connective and muscular), and soft tissues (such as ligaments, tendons, and in the menisci) (42–44, 101–103). Risk factors include aging, breed, obesity, joint injury, nutrition and genetics.

Currently, one out of five dogs or horses suffer from OA. Also, 52.5 million people in the US (10–12% of the adult population; 40% of individuals aged 50 years and over) and 250 million people worldwide have symptomatic OA (104–107).

Before discussing the anti-arthritic effects of HA, it is necessary to describe briefly the pathophysiology of OA. Pathophysiology of OA is very complex and it has been studied for decades, yet precise pathways and mechanisms have yet to be fully understood (42–44, 103, 108, 109). Horton et al. (100) described cellular, molecular, and matrix changes in OA. Aging and inflammation (inflammaging) and oxidative stress appear to be major contributing factors to the development and progression of OA (99, 110). Increases in proteolytic/catabolic enzymes (such as collagenases, aggrecanases, and matrix metalloproteinases), oxidative (superoxide and hydrogen peroxide) and nitrosative (nitric oxide and peroxynitrite) stress, inflammatory cytokines (IL-1β, TNF-α, and leptin), PGE_2_, cAMP, TSG-6, NF-κB, toll-like receptors (TLR), EP4 receptor, adiponectin, and many other molecular pathways (MAPK, NF-κB, TGFβ, Wnt/β-catenin, JNK, p38, NUDT7-PGAM1 axis, HIF-1α:CRAT:miR-144-3p axis dysregulation, and others) are involved in the pathogenesis of OA (42–44, 65, 109, 111–119). In several studies, some miRNAs have been implicated in development of OA (120–124), while others are involved in suppression of OA (125, 126).

Apoptosis (programmed cell death) of chondrocytes (chondroptosis) has also been reported to be a major factor in the degeneration and failure of articular cartilage in OA (127–132). The common molecular inducers of chondroptosis may include ROS, RNS, cytokines [IL-1β, TNF-α, TNF-related apoptosis-inducing ligand (TRAIL), and Fas ligand], and mechanical stress (131). In an *in vitro* study, Miwa et al. (133) demonstrated that chondroptosis appears to be due to elevated PGE_2_ through a cAMP-dependent pathway. In addition to this inflammatory pathway, mitochondrial dysfunction pathway leading to oxidative stress is another contributing factor to chondroptosis (134). Hashimoto et al. (135) and Kim et al. (129) reported that the collagen framework is important in the maintenance of chondrocyte survival in cartilage, and upregulation of Fas ligand in matrix depleted specimens suggested that the Fas pathway may have a role in chondroptosis induced by matrix depletion. In another study, Blanco et al. (127) found that IL-1-stimulated chondroptosis occurs due to excess generation of NO. Excess NO and ROS are reported to suppress mitochondrial activity (136, 137) and cartilage matrix synthesis, and enhance MMPs activity (131, 138). Of course, mitochondrial activity can also be modulated by IL-1β and TNF-α in chondrocytes (139). In late-stage OA, the cartilage becomes hypocellular, often accompanied by lacunar emptying, which has been considered as evidence that chondrocyte death is a central feature in OA progression. Chondroptosis is also characterized by increases in caspase-3 and−8, DNA fragmentation, increases in MMPs and ADAMTS, and decreases in aggrecan and type II collagen. It remains unclear whether chondroptosis is a cause or consequence of cartilage degeneration in OA (131, 140).

OA is characterized by the degradation of cartilage matrix components (including cartilage-specific type II collagen and the PG aggrecan), resulting in the loss of cartilage structure and function (42, 43, 141). ECM loss occurs due to elevated enzyme (collagenases, aggrecanases, and MMPs) activity. Chondrocyte death and ECM loss may form a vicious cycle, with the progression of one aggravating the other, and there appears to be a correlation between the degree of cartilage damage and chondroptosis (131).

Reversal of some of these mechanisms (as described above) provides the rationale for the use HA in OA.

#### HA Viscosupplementation, Mechanism of Action, and Pharmacological Effects

##### Rheological properties of HA and OA therapy

Rheological properties (such as MW, concentration, and viscoelasticity) of HA/HA derivatives (hereafter called HA) in formulation and route of administration are major determining factors for successful therapy of OA (10, 57, 142–145). The degree of anti-inflammatory, immunomodulatory, analgesic, and anti-OA effects of HA are also reported to be determined by the MW of HA and route of administration (25, 55, 78, 82, 107, 146–149).

Because OA affects a limited number of joints (such as shoulder, elbow, stifle, hip, hands and feet), local treatment, such as an IA HA delivery system, has been reported to be of paramount importance (150–152). IA administration is reported to be more effective than oral or IV because it avoids systemic exposure and potential adverse side effects. IA treatment with HA has been investigated in a number of studies, and it has been used for decades as OA therapy in dogs, horses, and humans (153–156). The concept of viscosupplementation, first proposed by Balazs and Denlinger (73), is based upon the hypothesis that IA injection of HA into OA joints could restore the rheological properties of the SF, promote the endogenous synthesis of a higher MW and possibly more functional HA, thereby improving mobility, and articular function, and decreasing pain.

Some studies revealed that HA with a MW larger than that of the injected solution were found in the SF of humans (72) and animals (71, 74). Although, HA concentrations remained within the physiological range. Since the concentration and MW of HA are significantly reduced in SF of OA (128, 164, 166, 184), viscosupplementation by IA injections of HA was recognized as a useful therapeutic option in the treatment of OA in different joints of several species (58, 66, 74, 157–159). In SF, HA is the major chemical component produced by synoviocytes, fibroblasts, and chondrocytes. Native HA has a MW of 4,000,000–10,000,000 Da, and is present in articular fluid in a concentration of about 0.35 g/100 mL (160). Within the normal adult knee, there is ~2 mL of SF, with a HA concentration of 2.5–4.0 mg/mL (158). In equines, the HA concentration in joints can be in the range of 0.33–1.5 mg/mL (161) and MW in the range of 2,000,000–3,000,000 Da.

In horses, the concentration of HA is reported to be significantly lower in arthritic joints (162). In OA, synovial HA is depolymerized (MW, 2,700–4,500 kDa) and cleared at higher rates (11–12 h) than normal (20 h) (27, 73, 158). An amphiphilic HA derivative was prepared by the amidation of the carboxylic group of the glucuronic acid called HYADD4-G (HY4). Borzacchiello et al. (74) compared viscoelastic properties of HY4 at a concentration of 5 mg/mL in phosphate buffered saline with solutions of native HA having the same MW. The addition of HY4 to equine SF increased its viscoelasticity at all the SF:HY4 ratios tested, thereby increasing the SF rheology presenting a new option in viscosupplementation therapy of OA. In sheep, IA HA is reported to be cleared from the acutely inflammed synovial joint within 20 h (157). These changes are known to reduce the viscoelasticity of the SF in OA (106, 163). It needs to be mentioned that SF HA concentration should not be used as a diagnostic biomarker of OA (164) since HA concentration can be influenced by other disease conditions.

Previous studies suggested that a more potent symptomatic effect was noted with higher-MW HA preparations, as compared to those of lower- or intermediate-MW HA (155, 165–167). Ghosh and Guidolin (25) demonstrated that HA with MWs in the range of 500,000–1,000,000 Da partially restored SF rheological properties and synovial fibroblast metabolism in animal models.

Tikiz et al. (168) compared the efficacy of IA injections of a lower-MW HA (Ostenil) with a higher-MW viscosupplement (Hylan-G-F 20, Synvisc) in hip OA. Findings revealed that both types of viscosupplementation produced a significant clinical improvement during the 6-months follow-up period, however, one formulation was not better than the other. In a similar study, Gigis et al. (77) reported that IA injections using higher-MW or lower-MW can improve joint function and reduce stiffness and pain in patients suffering from knee OA, however, no clear difference in benefits seems to exist between the two preparations and neither can slow disease progression based on radiological findings. Maheu et al. (159) compared the efficacy and safety of two different MW HA (F60027 and Hylan G-F20) in knee OA patients. Findings revealed that both preparations were equally effective in reducing functional impairment and relieving pain.

Bagga et al. (59) reported that the lower-MW HA preparations (500,000–1,500,000 Da) can achieve maximum concentration in the joint and are thought to reduce inflammation, however, they present lower elastoviscosity than native HA. Higher-MW preparations (6,000,000–7,000,000 Da) result in a better increase in fluid retention in the joint and possibly a stronger anti-inflammatory effect (66). In essence, efficacy might be related to rheological properties including MW and viscoelasticity of the preparation (169, 170). There is controversy over the efficacy of orally administered HA. Pharmacokinetic data revealed that orally administered higher-MW HA also reached the joint (82), which provides a rationale for the oral supplementation of HA.

From these studies, it can be concluded that viscosupplementation properties of HA therapy is more effective via IA than oral or IV administration, which can be due to augmentation of the lubricating potential of the SF in the OA joint (76). More studies need to be done to elucidate the role of MW of HA in viscosupplementation.

Mechanisms-based anti-OA effects of HA are summarized in Table 2.

**Table 2:** Mechanisms-based anti-OA effects of HA (some important studies).

**Specific effect**	**Mechanism(s)**	**Overall effect**	**Major reference(s)**
Antioxidative/Antinitrosative, and anti-inflammatory	HA reduced nitric oxide, superoxide, and hydroxyl radicals, PGE2, and diminished cell damage (*in vitro* and *in vivo* studies)	Antioxidative, antinitrosative, anti-inflammatory, chondroprotective, and anti-OA	(57, 65, 111, 127, 171–176)
	HA protected mitochondria from oxidative stress, and chondrocytes from apoptosis (*in vitro* study)	Antioxidative, anti-chondroptosis, protection of mitochondrial function, and anti-OA	(134)
	HA in combination with chondroitin sulfate reduced lipid peroxidation, restored GSH and SOD, decreased TNF-α, and reduced infiltration of activated neutrophils (*in vivo* study in rats)	Free radicals scavenging, Antioxidative, and anti-arthritic	(177)
	High MW HA suppresses MMPs and ADAMTS, and interacts with CD44, COX-2, and PGE2 (*in vitro* study)	Anti-inflammatory and anti-OA	(178)
	HA decreased PGE_2_ and increased cAMP in SF (*in vivo* human study)	Anti-inflammatory, anti-chondroptosis, and anti-OA	(133, 179)
	HA suppresses: (1) MMP-1 and MMP-3 in human synovial cells, and (2) MMP-1, MMP-3, and RANTES from chondrocytes via CD44 interaction (*in vitro* studies)	Anti-inflammatory, chondroprotective, and anti-OA	(180, 181)
	HA down-regulates aggrecanase-2, cytokines (TNF-α, and IL-8) and iNOS through interaction of CD44 in FLS (*in vivo* study)	Anti-inflammatory and anti-OA	(182)
	HA suppressed IL-1-α-induced PGE_2_ production in human OA synovial cells. The effect was dose- and MW-dependen t (*in vitro* study)	Anti-OA	(183)
	HA reduced IL-1-induced PGE_2_ and NO concentrations and decreased apoptosis in human OA chondrocytes (*in vitro* study)	Anti-nitrosative, anti-inflammatory, and anti-chondroptotic	(175)
	HA inhibits the expression of u-PA, PAI-1, u-PAR, and MMPs in chondral, meniscal, and synovial cultures of early OA (*in vitro* studies)	Anti-inflammatory and anti-OA	(167, 184)
Analgesic	HA relived joint pain by inhibiting PGE_2_ production (*in vitro* and *in vivo* studies)	Analgesic and protected cartilage degeneration	(15, 25, 185)
	Elastoviscous properties of HA (MW-dependent manner) are determining factors in reducing pain-nerve activity in normal and arthritic joints in rats, cat, dogs, horse, and sheep. Higher- MW HA with high concentration reduced pain by modifying the activity of mechanosensory channels (*in vivo* and *in vitro* studies)	Analgesic	(73, 76, 147, 186–188)
	HA produces reduction of the sensitivity of mechanosensory ion channels of nociceptive nerve terminals, using *Xenopus laevis* oocytes (*in vitro* study)	Analgesic	(189)
	Sodium HA of higher-MW attenuated the nociceptive responses in arthritis by inhibiting PGE_2_ and BK synthesis in the synovial joint of rats (*in vivo* study)	Antinociceptive	(69)
	HA decreased cytokines, leptin, and BK in SF and serum of OA patients (*in vivo* study)	Analgesic and anti-inflammatory	(190)
	In a dose-dependent manner, HA interacts with HA receptors on or surrounding the free nerve endings that detect pain in the joint tissue in rats (*in vivo* study)	Analgesic	(78)
Structure and function of cartilage and bone	HA ameliorated IL-1β-induced gene expression of matrix degrading enzymes (*MMP1, MMP13, ADAMTS5*), inflammatory mediators (*IL6, PTGS2*) by human OA chondrocytes and synovial fibroblasts. Inhibition of phosphorylation of the cell signaling molecules (JNK, p38, and NF-κB) (*in vitro* study)	Chondroprotective and anti-OA	(191)
	HA inhibits production of MMP-13 via CD44 and p38 in chondrocytes/articular cartilage (*in vitro* study)	Chondroprotective, cartilage repair, and anti-OA	(51, 192)
	HA inhibits expression of ADAMTS4 (aggrecanase-1) in human OA chondrocytes (*in vitro* study)	Chondroprotective, and anti OA	(193)
	Higher-MW HA inhibits cartilage degeneration and chondrocyte loss (*in vivo* studies)	Chondroprotective, cartilage protective, and anti-OA	(194–196)
	HA increases proteoglycan synthesis in bovine and equine articular cartilage (*in vitro* studies)	Cartilage repair	(172, 197)
	HA produces anti-chondroptosis by attenuating NO production (*in vivo* studies)	Anti-chondroptosis, and chondroprotective	(65, 198)
	HA downregulated MMP-3 and IL-1β, but not TIMP-1 expression (*in vivo* study)	Chondroprotective and cartilage repair effect	(67)
	HA inhibits PPAR-γ mRNA expression and exerts anti-chondroptosis	Protection of chondrocytes and cartilage degeneration	(199)
	HA regulates the function and distribution of sulfated GAG	Maintains normal structure and function of bone and cartilage	(200)
	HA suppressed IL-1β-induced-transcriptional activity of type α2(VI) collagen (*in vitro* study)	Chondroprotective	(201)
	HA exerts anti-Fas-induced apoptosis in human chondrocytes through interaction with CD44 and CD54 (ICAM1) (*in vitro* study)	Anti-apoptotic, and anti-cartilage matrix degradation in OA	(202)
	HA influences multiple receptors, proteins and signaling pathways; involved in ECM and inside the cell (*in vitro* study)	Cell adhesion, migration, and proliferation in maintaining cartilage homeostasis	(37)
	HA decreased synovial hypertrophy, macrophages, lymphocytes, mast cells, and adipocytes, and increased synovial FLC (*in vitro* and *in vivo* studies)	Cartilage repair effect	(25)
	HA increases RANKL expression in bone marrow stromal cells through CD44 receptor (*in vitro* study)	Improves bone metabolism	(203)
	HA inhibits expression of u-PA, PAI-1, u-PAR, and MMPs in synovial fibroblasts of OA (*in vitro* study)	Anti-OA	(184)
	Higher-MW HA downregulated MMPs and PA/plasmin expression in chondral, meniscal, and synovial cultures (*in vitro* study)	Delayed cartilage OA progression	(167)
Rheological properties of HA and SF	HA increased viscoelasticity, anti-inflammatory potential, increased proliferation of chondrocytes (*in vitro* and *in vivo* studies)	Anti-inflammatory, Chondroprotective, and anti-OA	(57, 74, 169, 170, 178, 204, 205)
	HA stimulated synoviocytes of high MW HA synthesis, and reduced synovial hyperplasia (*in vivo* studies)	Anti-OA in sheep	(206, 207)
	IA viscosupplementation promoted endogenous HA production in SF of OA knee. Increased HA concentration and viscoelasticity (*in vitro* and *in vivo* study)	Disease modifying effect in OA	(25, 59, 73, 75)
Pharmacokinetics of HA	After oral administration of ^99m^Tc-HA, it readily absorbed, distributed and excreted in rats and Beagle dogs	Rapid uptake, distribution and excretion of HA	(82)
	Elimination *t*$\frac{1}{2}$ of [^3^H]HA in rabbits and humans	Short half-life of HA in humans and animals	(94, 95)
	HA also distributes to lymphatic system and connective tissues	Rapid distribution and elimination of HA	(82–85)
	Metabolic half-life of HA in sheep SF is reported to be 27 h. Rapid elimination of HA from liver and blood.	Rapid absorption and elimination	(83, 157)

#### Antioxidative, Anti-inflammatory, and Analgesic Effects of HA

It is well-established that oxidative/nitrosative stress and inflammation are involved in OA-cartilage degeneration. In surgically-induced OA in New Zealand white rabbits, Takahashi et al. (65) found that the amount of nitric oxide (NO) produced by the meniscus was much greater than that produced by the synovium. Also, NO production in the meniscus and synovium of the HA group were significantly lower than those without HA treatment. The results suggested that the inhibition of NO production in meniscus and synovium might be a part of the mechanism of the therapeutic effect of HA on OA. In addition to NO (65, 111, 175), superoxide and hydroxyl radicals are also reported to be involved in the pathogenesis of OA, and their levels are reduced by HA treatment (111, 134, 171, 176, 177).

Philip (165), Ghosh and Guidolin (25), and Jerosch (155) reported that HA exerts pharmacologic actions by mitigating the activities of pro-inflammatory mediators and pain producing neuropeptides released by activated synovial cells (42–44, 154). These authors also described the interaction of HA with HA/pain receptors and analgesic effects. Moreland (14) noted that HA can reduce nerve impulses and nerve sensitivity associated with the pain of OA. HA can also reduce OA-associated pain by decreasing PGE_2_ and bradykinin (BK) synthesis, as well as substance P [Reviewed in (42–44)]. Gotoh et al. (78) found that HA with a MW >40 kDa produced an analgesic effect, and HA of 860 and 2,300 kDa produced high and long-lasting analgesia by interacting with HA receptors. Findings also suggested that the larger the molecule of HA, the higher the affinity of HA receptors for binding. Since HA did not directly bind to BK receptors, then analgesic effects of HA appears to be brought on by the interaction of HA and HA receptors on or surrounding the free nerve endings that detect pain in the joint tissue. Gomis et al. (147) found that the elastoviscous properties of HA solutions are determining factors in reducing pain-eliciting nerve activity both in normal and in inflammed rat joints.

In an *in vitro* study, Sasaki et al. (180) demonstrated that the expression of MMP-1 and MMP-3 mRNAs were induced by IL-1β in human synovial cells, and were strongly downregulated by HA (10 or 1,000 μg/mL). HA may inhibit binding of IL-1β to its membrane bound receptor by covering the cell surface, thereby suppressing the production of MMPs. The study suggested that IA HA may rescue inflammed joints from bone and cartilage destruction by reducing the production of MMP-1 and MMP-3. Yasui et al. (183) reported suppression of IL-1α-induced PGE_2_ production in human synovial cells by HA in a dose- and MW-dependent manner. In another *in vitro* study, Maneiro et al. (175) investigated the effects of two HA formulations (Hyalgan®, 500–730 kDa HA, Bioibérica SA; and Synvisc®, hylan of 6,000 kDa, Biomatrix Inc.) on PGE_2_ and NO concentrations and in human OA chondroptosis. Results revealed that 500–730 kDa HA (200 μg/ml) decreased the synthesis of both IL-1-induced PGE_2_ and NO by 70 and 45%, respectively. Both HA preparations at 200 μg/mL decreased chondroptosis (40 and 36%, respectively). HA has also been reported to protect against hydroxyl (OH·) radicals generation (171). It has been demonstrated that by modulating rheological properties (such as viscoelasticity) of HA and SF by employing HA of selective MW or HA-derivatives, anti-inflammatory activity can be greatly improved (57).

Hsieh et al. (167) investigated the effects of HA with different MWs on the expression of the plasminogen activator (PA)/plasmin system, urokinase-type PA (u-PA), PA inhibitor-1 (PAI-1), and MMP-2 and MMP-9 in a series of chondral, meniscal, and synovial cultures of early development of OA. Higher-MW HA (Synvisc) provided the greatest ability to inhibit MMP-2, MMP-9, u-PA and PAI-1 expression. Findings clearly demonstrated that the therapeutic effects of using higher-MW HA to treat early OA may be partially dependent on downregulation of the PA/plasmin system and MMPs expression, which delay the structural progression of OA.

In contrast to findings discussed above, Asari et al. (148) reported that lower-MW HA can enhance or induce inflammation through Toll-like receptor 4 (TLR-4). However, oral administration of higher-MW HA (900 kDa) to MRL-lpr/lpr mice modulates Th-1-type autoimmune disease and inflammation by up-regulating SOCS3 expression and downregulating pleiotrophin expression via TLR-4.

#### Chondroprotective, Anti-chondroptotic, and Cartilage Repair Effect

The chondrocyte is the unique resident cell of articular cartilage, which is responsible for ECM composition, regulation, and homeostasis (110, 208). Normally, joint cartilage consists of 5% chondrocytes and 95% ECM. The ECM of articular cartilage is comprised of complex networks of proteins and glycoproteins, all of which are expressed by chondrocytes. In OA, chondrocytes serve as mechanosensors and osmosensors, and their metabolism is influenced by microenvironment that in return influences ECM composition, organization and ultimately the mechanical resilience of cartilage (110, 209, 210). As OA progresses, chondrocytes lose their ability to maintain cartilage homeostasis, as a result of decline in mitotic and synthetic activity, to respond to anabolic growth factors, and to synthesize cartilage-specific PG core proteins (CSPCP) (211).

In regard to cartilage repair, Kuroki et al. (154) suggested that IA administration of HA has a direct effect on chondrocytes or synoviocytes and the production of transforming growth factor (TGF)-β, basic fibroblast derived growth factor (FGF), and insulin-like growth factor (IGF)-1. Histological evidence suggests that HA prevents the degradation of cartilage and may promote its regeneration. Ghosh and Guidolin (25) also provided evidence that HA treatment mitigated synovial hypertrophy and increased the numbers of synovial fibroblast-like cells (FLC) while decreasing macrophages, lymphocytes, mast cells, and adipocytes. HA appears to provide cartilage protection by the downregulation of cytokines, free radicals, and proteolytic enzymes in synovial fluid.

In an early *in vitro* study, Palmoski and Brandt (212) reported that PGs aggregation in severely OA affected femoral cartilage was impaired and showed no interaction between PGs and HA. Fukuda et al. (172–174) reported a stimulatory effect of HA on PG synthesis and prevented cartilage degradation in IL-1-induced cartilage destruction, probably acting as a free-radical scavenger. However, inhibition of IL-1-induced superoxide anion by HA does not appear to interfere with binding of IL-1 to chondrocytes. Whether HA inhibits superoxide anion in a receptor-mediated fashion, as does CD44 (47), remains yet to be elucidated.

Bagga et al. (59) examined the effect of IA Hylan GF-20 injections on synovial fluid HA concentration, viscosity, and elasticity over a 6-months period in patients with mild to moderate OA of the knees. The findings suggested that HA viscosupplementation appears to promote endogenous HA production, since the concentrations and MW of native HA declines in OA (73, 75).

Takahashi et al. (67) assessed the effects of HA (0.3 mL, once a week for 5 weeks) on MMP-3, IL-1β, and tissue inhibitor of metalloproteinase-1 (TIMP-1) gene expression in surgically-induced OA in New Zealand white rabbits. IL-1β is abundantly synthesized by OA synovium and cartilage (213), and induces the expression of MMP-3 (214) as well as PGE_2_ (215), and inhibits the formation of ECM (216). Takahashi et al. (67) suggested that one of the mechanisms of therapeutic effects of HA is down-regulation of MMP-3 (stromelysin) and IL-1β in synovium during early development of OA. TIMP-1 expression was not influenced by HA. In an *in vitro* study, using fluorescence microscopy, HA (800 kDa; 1 mg/mL) was shown to significantly suppress IL-1β-stimulated MMPs (MMP-1, MMP-3, and MMP-13) expression via CD44 surface receptor in human OA articular cartilage (51). Findings of this investigation revealed direct interaction between HA and CD44 receptor on chondrocytes.

In an *in vitro* study, Goto et al. (201) examined the effect of IL-1β on alpha2(VI) collagen gene in cultured rabbit articular chondrocytes and how this effect was reversed by HA. The chondrocytes cultured with IL-1β (1 and 10 ng/mL) showed significant decrease in alpha2(VI) collagen mRNA expression. However, the addition of both IL-1β and HA (1 mg/mL; MW 900–2,000 kDa) suppressed the reduced mRNA levels. Findings of this study suggested that suppression of transcriptional activity for type VI collagen appears to be associated with the reduction of cartilage matrix tissue and that HA is associated with the suppression of the effect of IL-1β. In another *in vitro* study, a hexadecylamide-derivative of HA (HYMOVIS®) provided better ameliorating effects than unmodified HA (MW 500–730 kDa) on human OA chondrocytes and synoviocytes, by inhibition of phosphorylation of the cell signaling molecules JNK, p38, and NF-κB.

In an *in vitro* study, Lisignoli et al. (202) isolated chondrocytes from human OA knee cartilage, and evaluated the effect of HA on both spontaneous and anti-Fas-induced apoptosis. HA (MW 500–730 kDa) at 1 mg/mL significantly reduced the anti-Fas-induced chondroptosis (by binding its specific receptors, CD44 and ICAM-1), but did not affect spontaneous chondroptosis.

From several other studies, it has also been suggested that HA inhibits: (1) IL-1β-induced MMPs activity in synovial fluid (68), (2) mRNA expression of proinflammatory cytokines, COX-2, and PGE_2_ production via interaction with CD44 receptors in subacromial synovial fibroblasts, (3) expression of ADAMTS4 (aggrecanase-1) in human OA chondrocytes (193), and (4) osteoclast differentiation via Toll-like receptor 4 (217).

### Experimental Studies (Rat, Rabbit, and Sheep)

#### Rat

In the context of OA and ameliorating effects of HA, the rat has mainly been used as a model for pain assessment (218). In the rat model of knee pain reaction (*in vivo*), Gotoh et al. (78) investigated the mechanism of the analgesic effect of sodium HA. The simultaneous administration of PGE_2_ with bradykinin (BK) or hyaluronidase digestion of endogenous HA produced hyperalgesia in BK-induced knee pain. Results revealed that higher sensitivity to the pain reaction is induced in a diseased joint (higher PGE_2_ content, lower concentration and MW of HA in SF) than in a normal joint. Sodium HA definitely decreased BK-induced pain, and its analgesic effect was observed for a longer period, depending on its dose in pre-treatment and the degree of its distribution in synovial tissues. Gotoh et al. (78) suggested that increasing viscosity of SF by increasing HA concentration could decrease pain even without normalizing MW in the joint. Sodium HA seems to exert its analgesic effect by blocking pain receptors in synovial tissues and holding endogenous pain substances in its molecule. It can be suggested that the characteristic steric configurations of higher-MW HA are needed for the manifestation of the analgesic effect. Furthermore, HA did not directly bind to BK receptors, indicating that analgesia of HA appears to be brought on by the interaction of HA and HA receptors on or surrounding the free nerve endings that detect pain in the joint tissue.

Gomis et al. (147) examined the effects of three different preparations of HA on the sensitivity of nociceptors in the normal and the acutely inflammed rat joint. Findings indicated that viscoelasticity and MW are the determining factors in reducing pain-eliciting nerve activity both in normal and in inflammed rat joints. Findings also revealed that the greater the viscoelasticity of the preparation, the fewer repeat injections were needed for long-lasting pain relief. Aihara et al. (69) investigated mechanisms of sodium HA with different MWs in monosodium urate (MSU) crystal-induced arthritis and nociception in rats. Results suggested that in a MW- and dose-dependent manner, sodium HA-induced antinociceptive effect may be due to the inhibition of PGE_2_ and BK synthesis in the synovial joints, thereby resulting in an the graded abnormal gait. In regard to mechanism of action of HA viscosupplementation, Gomis et al. (147) proved that the exchange of pathologic fluid of low elastoviscosity and low HA concentration with a pure HA solution of high viscoelasticity and high concentration produced an immediate decrease in the concentration of pain-stimulating and inflammatory agents in the joint. Also, the injection of a high concentration (10–20 times > than normal) of higher-MW HA into an arthritic joint results in a nearly normal IA fluid. The injected HA flowed out of the joint in 5–7 days, but the analgesic effect persisted longer.

In an experimental study in rats, Campo et al. (177) induced arthritis by intradermal injections of 250 μg of bovine type II collagen in 125 μl of 0.1 M acetic acid emulsified in an equal volume of complete Freund's adjuvant. Rats received GAGs (HA and chondroitin sulfate) in a volume of 1 mL/kg body wt, ip/day until the 20th day. HA and chondroitin sulfate (CS) reduced lipid peroxidation, restored the endogenous antioxidants (reduced glutathione and superoxide dismutase), decreased plasma TNF-α levels, and limited synovial neutrophil infiltration. The study results suggested that collagen-induced erosive damage in arthritis may be due to free radicals and can be treated with the antioxidative properties of GAGs. GAGs also exhibited anti-inflammatory property in rat arthritis model.

#### Rabbit

In an *in vitro* study, Goto et al. (201) investigated how the type α2(VI) collagen gene is regulated by IL-1β in cultured rabbit articular chondrocytes. To investigate the effect of HA on this collagen mRNA expression by IL-1β, chondrocytes were exposed to IL-1β (10 ng/mL) in the presence of HA (0.01, 0.1, 1 mg/mL; MW of 900 kDa). Chondrocytes were also exposed to IL-1β (10 ng/mL) in the presence of HA (1 mg/mL) with MWs of 200, 900, and 2,000 kDa. Findings indicated that suppression of transcriptional activity of type VI collagen is associated with the reduction of cartilage matrix tissue, and that HA is associated with the suppression of the effect of IL-1β.

In a number of experimental OA studies, HA of different MWs have been evaluated for therapeutic efficacy and safety in rabbits. Kikuchi et al. (195) examined the effect of HA (0.1 mL/kg) with varying MW formulations [800,000 Da, HA80 (1%); or 1,900,000 Da, HA190 (0.01–1%)] on cartilage degeneration in surgically-induced rabbit model (partial meniscectomy) of OA. With HA190 (1%), there was dramatic inhibition of cartilage degeneration in both the femoral condyle and the tibial plateau, and the degree of protection was greater than with HA80. Scanning electron micrographs of femoral cartilage showed that cartilage degeneration was less severe with HA190 than with saline. Findings also suggested that IA administration of higher-MW HA is more effective than lower-MW HA in inhibiting cartilage degeneration in early OA. Researchers from the same group administered sodium HA (MW 2,020,000, HA202) into the right knee of mature rabbits with osteoarthrosis (ACLT model), and found that the cartilage degeneration and chondrocyte loss were less compared to rabbits treated with a formulation of HA95 (MW 950,000) and even greater than the control (saline) group (194). In another study, Shimizu et al. (196) injected IA HA of varying MW in surgically-induced OA New Zealand white rabbits, once a week for 5 weeks in one knee, using the other knee as a control. Histomorphometric analysis revealed that HA-treatment suppressed cartilage degeneration. However, biochemical analysis of synovium showed no significant difference between treated and control.

Using the ACLT model, Amiel et al. (219) investigated the long-term effects of single and repeated administration of HA (Hyalgan®) therapy on OA progression. Findings revealed that repeated HA injections reduced the degree of articular degeneration, and may be advantageous in the long-term management of OA. Mainil-Varlet et al. (220) evaluated therapeutic efficacy of HYADD® 4-G (a HA derivative) alone or in conjunction with growth factors. Results suggested that HYADD® 4-G delayed cartilage degeneration and that the association of HYADD® 4-G with growth factors was synergistic. IA HA has also been shown to act via several disease modifying mechanisms in OA cartilage and synovium, but the effect of HA on subchondral bone in patients with OA remains unclear (65).

In a rabbit model of OA (partial meniscectomy), Hashizume et al. (15) demonstrated that higher-MW HA inhibited PGE2 production, protected cartilage degeneration, and exerted an analgesic effect. In a mechanistic study on OA in New Zealand white rabbits, Zhou et al. (199) injected 0.3 mL sodium HA (1%) once a week for 5 weeks, 5 weeks post-ACLT. Eleven weeks post-surgery, the cartilage changes in medial femoral condyles revealed that the expression of peroxisome proliferator-activated receptor gamma (PPAR-γ) mRNA in saline-treated rabbits was greater than in HA-treated rabbits. Since PPAR-γ is associated with the chondroptosis (221, 222), HA may provide anti-chondroptotic and a protective effect on articular cartilage degeneration by inhibiting PPAR-γ mRNA expression. This can be one of the therapeutic mechanisms of HA in OA.

#### Sheep

In a pharmacokinetic study, Fraser et al. (157) estimated the metabolic *t*${}_{\frac{1}{2}}$ of HA (27 h) in SF in sheep from the rate of appearance of ^3^H_2_O in plasma after injection of highly polymerized labeled HA. This material is substituted with ^3^H in its acetyl group and is rapidly and almost completely degraded in sheep and other species to yield ^3^H_2_O. The study indicated that mild acute inflammation can induce major changes in the metabolic turnover of SF HA. Mean SF volume in the normal sheep hock joint was estimated to be 1.54 mL, and the concentration and content of HA were 0.54 mg/mL and 0.84 mg, respectively.

Ghosh et al. (223) evaluated two IA HA preparations (MW 800,000, AHA; and MW 2,000,000, DHA) in ovine joints with early OA (surgically induced medial meniscectomy). Animals when injected IA with 1 mL (10 mg/mL) of either HA preparation once a week for 5 weeks beginning at 16 weeks after initiation of arthropathy. Meniscectomy of sheep stifle (knee) joints resulted in matrix changes indicative of early OA. Both HA preparations appeared to improve gait (Ground Force Plate analysis), suggesting decreased lameness. However, cartilage damage (radiographic evidence) with DHA was found to be higher than with AHA. In a similar study, Ghosh et al. (224) examined the effects of AHA and DHA on cartilage composition and PG metabolism. The cartilage was analyzed for collagen and PG content and differential extractability with guanidine hydrochloride (GuHCl) solutions. The release of ^35^S-PGs from the tibial cartilage of the DHA-treated animals was found to be higher than in the saline-treated group. The biosynthesis of ^35^S-PGs, determined *in vitro*, for cartilage derived from the medial compartment was generally lower than for the lateral regions of the meniscectomized joints. The biosynthetic activity was further reduced in joints injected with the two HA preparations, but DHA reduced ^35^S0_4_ incorporation into PGs more than AHA. The authors concluded that reduced biosynthesis of ^35^S-PGs and secretion into media was a consequence of increased loading of joints in the HA-treated animals rather than a direct effect of these preparations on chondrocyte metabolism.

### Clinical Studies

#### Canine

Most studies in dogs with HA have been carried out in the surgically (anterior cruciate ligament transection, ACLT)-induced OA model. ACLT produces metabolic, biochemical, biomechanical and morphological changes in articular cartilage of the unstable knee that are consistent with those of OA in humans (225–231). Following ACLT, OA is often characterized by marginal osteophytes, fibrillation, cartilage PG depletion, synovial inflammation, and joint effusion (232). In the ACLT-induced OA model, the structure of newly synthesized [^35^S]-PG and its turnover have been characterized (225, 228). Arthropathy may release soluble protein(s) from the synovium, which may cause synovial proliferation and excessive secretion of HA, in addition to the concentration of water content and uronic acid (a PG metabolite).

Smith et al. (233) investigated the effects of HA (MW 1,500,000 Da) in ACLT-induced OA in mongrel dogs (5 weekly injections beginning the day after surgery), and compared results with saline-treated dogs. The severity of pathological changes in OA was graded, and composition of the cartilage and extent of aggregation of PG synthesized *in vitro* by cartilage slices were determined. The size and number of osteophytes and the extent of fibrillation of the articular cartilage in the unstable knee of the dogs that had received HA injections were grossly indistinguishable from those in the dogs whose knee had been injected with saline. In the saline-treated group, some dogs exhibited mechanical damage in the central region of the medial femoral condyle (MFC_c_) with deep cartilage ulcer, which extended down to the underlying bone. Other dogs in this group exhibited only pitting and fibrillation of the cartilage at this site. Also, in OA cartilage from the saline-treated group, the mean concentration of uronic acid was 30–60% greater than that in the contralateral knee. In contrast, the concentration of uronic acid in OA cartilage from the HA-treated dogs was 10–30% lower than that in cartilage from the contralateral knee. The PG concentration of cartilage in the OA knee was significantly reduced, suggesting that HA therapy might adversely affect the biomechanical properties of the cartilage.

In surgically-induced OA in dogs, biochemical and morphological changes in cartilage and the effects of IA injections of HA have been evaluated (234, 235). Abatangelo et al. (235) and Schiavinato et al. (236) reported beneficial effects of HA on the cartilage in response to damage, and a clear-cut inhibitory effect on the development of the FLC layer on the cartilage in untreated joints was noted. Marshall et al. (146) reported that a series of 3 weekly injections of 0.5 ml (4 mg) of HA (MW 6,000,000 Da) ameliorated the severity of OA in dogs. In addition to its contribution to the structural properties of the ECM, HA may have a role in regulating the synthesis of PGs during maturation of articular cartilage and in repair processes (154).

Both in preclinical and clinical studies, Flex choice, a novel patent pending nutraceutical product developed by Clinic-Choice, which contains Flexpro MD (low-MW HA, pure krill oil, and astaxanthin) has clearly shown an anti-OA effects in dogs. Pak et al. (237) demonstrated that Flexpro MD markedly reduced phosphorylation of NF-κB p65 and inhibition of κB-α (IκB-α) induced by lipopolysaccharides (LPS). In addition, Flexpro MD displayed anti-inflammatory effects in mice against LPS-induced arthritis by significantly reducing the expression of pro-inflammatory cytokines and other inflammatory markers, thereby reducing pain.

#### Equine

Since the early 1970's, IA HA has commonly been used in the treatment of synovitis and OA (knee, carpal, and fetlock joints), and osteoarthrosis in racehorses and heavy work horses (238–241). HA can be injected directly into an affected joint. Auer et al. (242) reported that in naturally occurring and experimentally induced OA in horses, IA injection of HA (40 mg) significantly reduced lameness and increased weight bearing on the treated limb, measured using the Ground Force Plate (GFP). HA is especially indicated for mild to moderate levels of synovitis associated with equine OA. However, it has limitations in treating severe synovitis or OA.

In an earlier study, Rose (239) injected IA HA in 16 horses with osteoarthrosis of the carpus, fetlock, hock, and coffin joints. In 11 of those horses lameness was completely relieved. The other five horses showed less swelling of the joint capsule, and increased range of joint movement. Rydell et al. (71) injected HA in combination with methyl-prednisolone or methyl-prednisolone alone in the joints of track horses with traumatic arthritis, and found that the clinical response was considerably better than when the corticosteroid was used alone. Using GFP, Gingerich et al. (186, 243) demonstrated that IA administration of 20 or 40 mg HA (in a dose-dependent manner) in experimentally induced OA in horses produced significant functional improvement in joints, while 5 or 10 mg did not. In a clinical trial, Philips (165) compared the efficacy of IA sodium HA products (Synacid, Equron, Hyalovet, Hyvisc, and Hylatrin V) in horses. These products vary in method of production, MW, solvent, and packaging. The MWs of the various sodium HA products are: 150,000 Da for Synacid; 1,500,000–2,000,000 Da for Equron; 750,000–1,000,000 Da for Hyalovet; 2,100,000 Da for Hyvisc; and 3,500,000 Da for Hylatrin (81, 241). Findings of this and other studies suggested that: (1) more than one treatment might be helpful for all these products, and (2) the effect appears to be MW- and concentration-dependent (164, 241).

McIlwraith (161) reported that sodium HA may affect the composition of SF through steric exclusion of active plasma components and leucocytes from the joint cavity by modulating chemotactic response. These investigators also studied molecular interactions between HA and CD44 receptors (present on lymphocytes, neutrophils, and synoviocytes) in horses. Recently, Niemelä et al. (244) reported analgesic and pain reducing effects of IA non-animal stabilized HA (NASHA) in the treatment of lameness localized in the metacarpophalangeal joint in horses. 67% of NASHA (3 mL)-treated horses were able to perform normal exercise, and scores in the flexion test improved compared to the saline -treated placebo group.

During the last decade, the use of IV HA has become more common, especially for less localized OA disorders. However, there is only one licensed product, made by Bayer, named Legend® in the US, and Hyonate® elsewhere. It has been suggested that when HA is used in combination with corticosteroids, HA not only improves beneficial effects of corticosteroids, but it can placate the side effects of certain corticosteroids.

In several other studies, HA in different forms and formulations has been found effective in ameliorating OA and other forms of joint ailments by analgesic, antioxidative/antinitrosative, anti-inflammatory, and cartilage repair effects by multiple biological and pharmacological mechanisms (186, 243, 245–249).

According to Canadian regulations, HA in HY-5-preparation should not be administered to horses that are to be slaughtered for meat.

### Humans

Currently, many formulations of HA are prepared with lower-MW (500–730 kDa), intermediate-MW (800–2,000 kDa), and higher-MW (~6,000 kDa) for IA injections and are available worldwide (250). Therapeutic efficacy of these products can differ based on HA origin, method of production, treatment schedule, MW, viscoelasticity and other rheological properties, half-life within the joint, and pharmacokinetics and pharmacodynamics. In general, the half-life of HA products in the joint is reported to be much shorter than their duration of clinical effect (251). Some of the FDA approved HA products include: Hyalgan (Fidia Pharma), Supartz (Bioventus), Orthovisc (DePuy Mitek), Euflexxa (Ferring Pharmaceuticals), Synvisc/Synvisc One (Sanofi-Aventis), and Gel-Syn (Institut Biochimique). In clinical studies, there is no evidence of the superiority of any one brand of viscosupplement over another brand. In general, patients below 65 years of age and those with less severe OA are likely to be benefited from HA viscosupplementation (252). Also, some clinical studies indicated that therapy with an IA HA formulation with a higher-MW (e.g., hylan G-F 20) was more effective in relieving OA-associated pain than therapies with lower-MWs (e.g., sodium hyaluronate) (142, 253–255).

In a large number of clinical trials, HA has been found to exhibit anti-OA effects. HA is a slow-acting anti-OA agent that can be used prophylactically or therapeutically as an anti-inflammatory symptom modifying and disease modifying agent (154, 156, 256–259). Evidence for disease-modifying activity of HA stems from: (1) the complex cellular and molecular effects of HA in the synovium and ECM of the articular cartilage, including interactions between exogenously administered HA and articular cartilage, subchondral bone, matrix PGs, and collagens; (2) the effects of HA administration in animal models of OA, including total or partial meniscectomy and ACLT; and (3) results of clinical trials using one HA, Hyalgan (sodium HA, MW 500–730 kDa) that evaluated structural outcomes, such as joint-space width, chondrocyte density and vitality, and arthroscopic evaluation of chondropathy (256). In a few clinical trials, IA injections of HA was found to inhibit joint space narrowing on X-ray images and slow the progression of cartilage degeneration in follow-up arthroscopy (260, 261).

Berenbaum et al. (250) found that treatment with an intermediate-MW (800–1,500 kDa) HA (GO-ON, Rottapharm) was significantly superior to lower-MW (500–730 kDa) HA (Hyalgan, Fidia Pharma) for knee OA symptoms. Findings revealed that a higher proportion of patients with treatment response was observed with GO-ON than with Hyalgan (73.3% vs. 58.4%). In a randomized, multicenter clinical trial, higher-MW HA (Orthovisc) was reported to be very effective and safe in the treatment of mild to severe knee OA. However, in other studies conducted with HA of different MW in knee OA patients, it has been concluded that higher MW HA preparations are not superior to intermediate- or lower-MW compounds (159, 262–267).

In a clinical study, oral administration of HA (200 mg/day) for 12 months improved the symptoms of knee OA in patients aged 70 years or younger when combined with quadriceps strengthening exercise (268). In another clinical trial, Nelson et al. (190) reported that oral intervention with HA (Oralvisc®) provided OA improvement. Findings revealed that the regulation of the inflammatory milieu detected both in serum and SF in the patients treated with an oral preparation has been associated with a trend toward a normalization of the HA turnover in SF. Furthermore, this partial normalization could suggest a slower OA progression since HA turnover has been associated with disease severity in preclinical models.

HA has been reported to produce some side effects, such as muscle pain, cramping, pain in the injected knee, and swelling in arms and legs making movement difficult (269).

### HA in Wound Healing

Wound healing is a complicated multi-step process, which involves various cell functions, such as cell migration, proliferation, basement membrane regeneration, and the formation of granulation tissue. According to Weigel et al. (270), the wound itself is a transitory organ or structure whose function is to remodel, successively, a series of increasingly complex and ordered ECMs. Because of its high concentration in skin and mucosa, HA is reported to have important biological roles in skin or mucosal wound healing by influencing inflammation, granulation, and reepithelialization (7, 10, 38, 39, 271–275). The level of HA is reported to be high in granulation tissue during the wound healing process. Using immunohistochemical techniques, Picker et al. (276) localized epithelium-, leucocyte-, and fibroblast-specific CD44 receptors for HA binding. In normal connective tissue and granulation tissue, CD44 staining was restricted to cells, whereas HA was diffusely distributed throughout tissues. In normal mucosa, CD44 was localized in all layers of the stratifying epithelium except for stratum corneum, and throughout the connective tissue. Electron microscopic examination of the basal and spinous cell layers displayed HA, both associated with the cell surface and free in the intercellular space. Oksala et al. (272) found that HA and CD44 were localized in the same region of the epithelium (around mucosal keratinocytes) in all stages of wound healing. Wang et al. (277) also noted that in the keratinizing skin epithelia both HA and CD44 receptor showed an intense staining with a close co-distribution. Kaya et al. (278) reported that the two major functions of CD44 receptors in skin may be the regulation of keratinocyte proliferation in response to extracellular stimuli and the maintenance of local HA homeostasis.

Oksala et al. (272) investigated the expression of PGs and HA during healing of human mucosal wounds. Findings revealed that CD44 surrounded migrating keratinocytes at all stages of wound healing. In epithelium, CD44 and HA remarkably localized to the same region. Expression of syndecan-1 was switched from the suprabasal cell layer of unwounded epithelium to the basal cell layer of the migrating wound epithelium. The area under the wound epithelium containing newly synthesized collagen fibers first became positive for decorin on day 7, where staining for biglycan was negative. Granulation tissue was also strongly positive for CD44 and HA. Migrating keratinocytes expressed both CD44 and syndecan-1. During differentiation of keratinocytes, expression of CD44 preceded that of syndecan-1. Binding of HA is probably not the only function of CD44 in keratinocytes. CD44 is a multifunctional cell surface PG that binds high endothelial venue ligand, HA, fibronectin, laminin, and collagens (272). The study concluded that different basement membrane-associated heparin sulfate proteoglycan (BM-HSPG) have multiple functions in keratinocyte migration and differentiation during reepithelialization.

In an interaction study, Weigel et al. (279) proposed that fibrin and HA are macromolecular regulators during inflammation and wound healing. These authors found significant binding of ^125^I[HA] with human, sheep, rabbit, dog, baboon, goat and pig fibrinogens, and no interaction with horse, rat, or cow fibrinogens. HA and fibrin have organizational, structural and regulatory functions (at both the molecular and cellular level) at different times during the wound healing process by exerting anti-inflammatory activity and new blood vessel formation (274, 280). Aya and Stern (281) reported that levels of higher MWHA are more prominent in the earliest stages of wound repair. However, in a number of experimental studies, higher-MW HA has been shown to inhibit angiogenesis, and lower-MW HA has been shown to promote angiogenesis (39, 282–284). The higher MW-HA polymers suppress angiogenesis with their ability to inhibit early response genes, such as *c-fos, c-jun*, and *Krox-20* in endothelial cells (285). Findings revealed that fibrin is a common component of the normal ECM and fibrin and fibrinogen specifically interact with HA. The early appearance of higher-MW HA and the later appearance of lower-MW HA at the wound site as it is degraded provides a mechanism to regulate and integrate the timing of the cellular activities needed to initiate and sustain the inflammatory response and the wound healing process (270, 286). This study also emphasized that thrombin-induced formation of fibrin clots is also affected by HA, which decreases the lag time before clotting and increases the rate of clot formation. It is noteworthy that an increase in circulating HA levels could adversely affect hemostasis and increase the risk of thrombosis or bleeding.

In conclusion, the wound healing process can be organized into three main events, and each event involves HA: (1) a matrix rich in HA is laid down in a cell-poor space, (2) mesenchymal cell migration is stimulated and the HA matrix is infiltrated by cells migrating from the adjacent tissues, and (3) cells within the HA matrix secrete both hyaluronidase, which degrades the HA, and sulfated GAGs and collagen, which concomitantly replace the HA and the matrix is remodeled (270, 281, 287).

### HA in Ophthalmic Conditions

The use of HA has been implicated in various ophthalmic conditions, including keratoconjuctivitis sicca (KCS) or dry eye disease (DED) in humans and animals (9, 10, 288–293). HA solutions (0.1% HA) effectively lubricate the ocular surface and are used for the relief of dry eye related symptoms (294). However, HA undergoes rapid clearance due to limited adhesion, which necessitates frequent instillation. Furthermore, highly viscous artificial tear formulations with HA blur vision and interfere with blinking. Lee et al. (294) synthesized a heterobifunctional polymer-peptide system with one end binding HA while the other end binds either sialic acid-containing glycosylated transmembrane molecules on the ocular surface epithelium, or type I collagen molecule within the tissue matrix. These investigators treated rabbit ocular surface tissues with binding peptides and HA solutions and demonstrated superior lubricating with long-lasting effect, and reduced kinetic friction coefficients compared to tissues treated with conventional HA solution. HA ameliorates the symptoms of DED by reducing irritation, and by moisturizing the eye, enhancing tear film stabilization, reducing friction during blinking, preventing binding of harmful substances to the eye, and replenishing the deficiencies of sodium HA in the tear film (293, 295). In a number of studies, HA has been shown to improve healing of corneal epithelial abrasion and wounds (296–302). Currently, Remend® (a product of Bayer) containing 0.4% Hyasant-S (a cross-linked, modified HA) is available to maintain lubrication and hydration of the eye in DED in dogs and cats.

HA plays an important role in ophthalmic surgery during operations of the anterior segment of the eye, such as trabeculectomy, cataract removal, glaucoma treatments, refractive surgery, and corneal plastic surgery (293). Due to its viscoelastic, lubricating, cushioning, hydrating, and other rheological properties, HA has been shown to facilitate tissue healing (such as corneal epithelium cell proliferation, cell migration, retinal reattachment, etc) following ophthalmological surgery (288, 293, 296, 299, 303–305). HA exerts its effects through the stabilization of the tear film, decreased duration of healing, minimized risk of adhesions, decreased formation of free radicals, and normalization of intra-ocular pressure. Taken together, HA appears to play significant roles in health and diseases of eye.

### HA in Chemoprevention and Cancer Therapy

In animal and cell models, HA has been implicated in tumor progression, and it has been reported to have a profound impact on several signaling pathways involved in cancer (10, 306, 307). The effects of HA are mostly mediated by interactions with cell surface receptors (CD44, RHAMM, LYVE, and TLR-2 and TLR-4) that, when activated, bind and respond to HA (308, 309). HA is the primary ligand for CD44 that is overexpressed in many cancer types including pancreatic, breast, lung, ovarian, prostate, etc (307, 308, 310–316). Members of the CD44 family of transmembrane glycoproteins emerge as major signal transduction control units (317). CD44 isoforms participate in several signaling pathways ranging from growth factor-induced signaling to Wnt-regulated pathways. The role of CD44 family members in tumor progression and metastasis is most likely linked to the function of the various isoforms as signaling hubs. Increasing evidence suggests that these proteins are directly involved in tumor and metastasis initiation. Since HA influences oncogenic signaling pathways, researchers found efficacious methods of inhibiting HA-receptor interactions in a manner that interferes with tumor progression. Treatment with small oligomers of HA that compete for constitutive binding of polymeric HA to its endogenous receptors appears to be one of the approaches. The hypothesis underlying this approach is that signal transduction by endogenous HA is dependent on high affinity, multivalent interaction with receptors and that small oligomers that bind monovalently will act as antagonists by replacing multivalent and cooperative interactions with low affinity, low valency receptor interactions. It has been reported that HA composed of 6–18 sugar residues binds monovalently to CD44, whereas larger polymers bind multivalently (318). Yang et al. (55) reported that native high MW HA (nHA) binding to CD44 selectively induces CD44 clustering, which could be inhibited by oligosaccharides of hyaluronan (oHA). Findings also revealed that HA regulates cell adhesion in a manner specifically dependent on its MW. oHA promoted cell adhesion, while nHA showed no effects.

HA-receptor interactions influence the activity of several signaling pathway components, including those that promote cell growth, survival, and motility, e.g., ErbB2/ErbB3, Ras, Erk, Src, NFκB, and phosphoinositide 3-kinase, depending on the type and physiological state of cells (306). These investigators have also shown that HA constitutively regulates activation of several receptor tyrosine kinases, such as ErbB2, EGFR, PDGFR, IGFR and c-MET, which are known to be important in progression of various cancer types. These events can lead to inhibition of downstream cell survival and proliferation pathways, e.g., phosphoinositide 3-kinase activity and phosphorylation of Erk, Akt, GSK3, BAD and FKHR (319, 320). HA oligomers competitively block binding of endogenous HA polymer to CD44, consequently giving rise to attenuated signaling. Misra et al. (321) showed that both constitutive activation of ErbB2 and ligand-mediated activation of IGF1R-β and PDGFR-β are reversed by co-treatment of the cancer (colon, prostate, and breast) cells with a HA antagonist, and concluded that HA serves a general function in receptor tyrosine kinase (RTK) activation.

The conjugation of a drug to HA can confer specificity and selectivity for cancerous cells, and can also provide a pharmacological advantage in terms of solubilization and stabilization. Montagner et al. (307) evaluated the efficacy of a loco-regional intraperitoneal treatment with a bioconjugate of HA (ONCOFID-S) delivered by chemical linking of SN-38, the active metabolite of irinotecan (CPT-11), to HA in a mouse model of ovarian carcinogenesis. Findings revealed that SN-38 conjugation to HA significantly improved the profile of *in vivo* tolerability and enhanced therapeutic efficacy for ovarian cancer treatment. *In vitro* results suggested that the conjugate selectively interacted with ovarian cancer cells through the CD44 receptor, disclosed a dose-dependent tumor growth inhibition efficacy comparable to that of SN-38 drug, and inhibited topoisomerase I function leading to apoptosis by a mechanism involving caspase-3 and−7 activation and PARP cleavage. Studies have also demonstrated that a derivative of HA and different types of HA-paclitaxel conjugates have a greater potential as anticancer therapies (322–325).

Taken together, the use of HA alone or in combination with established agents (doxorubicin, taxol, vincristine, methotrexate, imatinib, gemcitabine, cisplatin, 5-fluorouracel, etc.) seems to be a promising new avenue for anticancer therapeutics (306, 326–329).

## Toxicity and Safety Evaluation

HA, being a physiological component, is not expected to produce adverse reactions even after repeated administration (66, 330). In a large number of clinical trials, HA has been found to be safe and well-tolerated in OA patients, when given IA (107, 166, 251, 331–334). A number of studies concluded that lower-MW, intermediate-MW, and higher-MW HA are clinically safe and well-tolerated (159, 262–267).

Maheu et al. (159) observed 470 adverse events reported by 123 patients (88.5%) in the F60027 (Structovial®) group; and 492 adverse events reported by 122 patients (87.1%) in the Hylan G-F-20 (Synvisc®) group. A total of 102 treatment emergent adverse events (TEAE) were reported by 56 patients (40.3%) in the F60027 group and 120 TEAE were reported by 60 patients (42.3%) in the Hylan G-F20 group. In this investigation, six serious adverse events (SAE) were reported by 6 patients, 1 in the F60027 group and 5 in the Hylan group. Following IA injections, tolerance of HA was found to be satisfactory (1 in the F60027 group and 5 in the Hylan group).

Following IA HA administration, some minor side effects may occur, such as pain at the injection site (in 1–30% of patients), local joint pain and swelling (in 1–30%), and local skin reactions (in 3–21%) (106). Also, some forms of HA may cause these adverse effects more frequently than others. These effects are transient. In rare cases, treated joints may become infected (335).

## Contraindications

HA treatment is contraindicated in individuals who are hypersensitive to HA products, woman who are pregnant or nursing, pediatric patients, patients with bacteremia, or patients with infections in or around the target knee (106). Also, patients with liver or kidney diseases or Shar-pei dogs should not receive HA parenterally, as high circulatory HA levels may cause thrombosis.

## Concluding Remarks and Future Perspective

HA is a naturally occurring non-sulfated GAG produced in many body organs and tissues. HA is also produced by microbial fermentation. The applications of HA depend on its rheological properties (such as MW, viscoelasticity, etc) and therefore quality products are prepared having HA of specific MWs. HA has been considered to be a stealth molecule for joint health, as it plays multiple roles including: articular cartilage lubrication, antioxidative/antinitrosative, analgesic, anti-inflammatory, chondroprotective, prevent ECM degradation, and cartilage repair effects. HA exerts anti-OA effect by interacting with receptors, enzymes, and many other biomolecules. All these physicochemical and biological properties of HA appear to be MW-dependent. Thus, a rationale for HA in the use of OA or skin conditions exists. Future studies need to identify uses of HA in health conditions other than OA, and clarify the relevance of its MW, and long-term clinical trials in canine and equine.

## Author Contributions

All authors listed have made a substantial, direct and intellectual contribution to the work, and approved it for publication.

### Conflict of Interest Statement

RL and AjS are employees of Vets Plus Inc. The remaining authors declare that the research was conducted in the absence of any commercial or financial relationships that could be construed as a potential conflict of interest.

## Abbreviations

ACLT: anterior cruciate ligament transection
ADAMTS: a disintegrin and metalloproteinase with thrombospondin motifs
BK: bradykinin
MB-HSPG: basement membrane-associated heparan sulfate proteoglycan
cAMP: cyclic adenosine monophosphate
COX-2: cyclooxygenase-2
CSPCP: cartilage-specific PG core proteins
ECM: extracellular matrix
FDA: Food and Drug Administration
FLC: fibroblast-like cells
FLS: fibroblast-like synoviocytes
GAG: glycosaminoglycan
GSH: reduced glutathione
HA: hyaluronic acid/hyaluronan
HARE: hyaluronic acid receptor for endocytosis
HAS: hyaluronan synthase
IA: intra-articular
ICAM1: Inetercellular adhesion molecule 1
IGF: insulin-like growth factor
IL-1β: interleukin-1β
iNOS: inducible nitric oxide synthase
IV: intravenous
kDa: kilo Dalton
LYVE-1: lymphatic vessel endothelial hyaluronan receptor-1
MFC_c_: medial femoral condyle
MMP: matrix metalloproteinase
MSU: monosodium urate
MW: molecular weight
NASHA: non-animal stabilized HA
NF-κB: nuclear factor-kappaB
NO: nitric oxide
OA: oateoarthritis
PA: plasminogen activator
t-PA: tissue-type plasminogen activator
uPA: urokinase-plasminogen activator
PG: proteoglycan
PGE2: prostaglandin E2
PPAR-γ: peroxisome proliferator activated receptor-γ
RANTES, regulated on activation: normal T cell expressed and secreted
RHAMM: receptor for hyaluronate-mediated motility
RNS: reactive nitrogen species
ROS: reactive oxygen species
RTK: receptor tyrosine kinase
SAE: serious adverse events
SF: synovial fluid
SOD: superoxide dismutase
TEAE: treatment emergent adverse events
TGF-β: transforming growth factor β
TIMP-1: tissue inhibitor of metalloproteinase-1
TLR: toll-like receptor
TNF-α: tumor necrosis factor-alpha
TRAIL: TNF-related apoptosis-inducing ligand
TSG6: tumor-stimulated gene 6.
